# Development and evaluation of a novel antibody-photon absorber conjugate reveals the possibility of photoimmunotherapy-induced vascular occlusion during treatment *in vivo*

**DOI:** 10.18632/oncotarget.25831

**Published:** 2018-07-31

**Authors:** Yuya Isoda, Wen Piao, Eri Taguchi, Junko Iwano, Shigeki Takaoka, Aiko Uchida, Kiyomi Yoshikawa, Junichi Enokizono, Emi Arakawa, Kazuma Tomizuka, Yasuhisa Shiraishi, Kazuhiro Masuda

**Affiliations:** ^1^ Research Functions Unit, R&D Division, Kyowa Hakko Kirin Co., Ltd, Machida-shi, Tokyo, Japan; ^2^ Translational Research Unit, R&D Division, Kyowa Hakko Kirin Co., Ltd, Suntou-gun, Shizuoka, Japan; ^3^ Fuji Research Park, R&D Division, Kyowa Hakko Kirin Co., Ltd, Suntou-gun, Shizuoka, Japan

**Keywords:** antibody-photon absorber conjugate, EpCAM, photoimmunotherapy, vascular occlusion, targeted cancer therapy

## Abstract

Photodynamic therapy (PDT) utilize a photosensitizing agent and light for cancer therapy. It exerts anti-cancer effect mainly by inducing vascular occlusion at the irradiated site. By controlling the irradiation area, PDT can be used in a tumor-specific manner. However, the non-specific cellular damage in the surrounding normal tissue is still a serious concern. Photoimmunotherapy (PIT) is a new type of targeted cancer therapy that uses an antibody-photon absorber conjugate (APC). The superiority of PIT to PDT is the improved target specificity, thereby reducing the damage to normal tissues. Here, we developed a novel APC targeting epithelial cell adhesion molecule (EpCAM) as well as a negative control APC that does not bind to the EpCAM antigen. Our *in vitro* analysis of APC cytotoxicity demonstrated that the EpCAM APC, but not the negative control, was cytotoxic to EpCAM expressing COLO 205 cells after photoirradiation, suggesting that the cytotoxicity is antigen-dependent. However, in our *in vivo* analysis using a mouse xenograft tumor model, decreased volume of the tumors was observed in all the mice treated with irradiation, regardless of whether they were treated with the EpCAM APC or the negative control. Detailed investigation of the mechanism of these *in vivo* reveal that both APCs induce vascular occlusion at the irradiation site. Furthermore, the level of vascular occlusion was correlated with the blood concentration of APC, not the tumor concentration. These results imply that, similar to PDT, PIT can also induce non-targeted vascular occlusion and further optimization is required before widespread clinical use.

## INTRODUCTION

Various antibody drug conjugates (ADCs), which are formed by linking a cytotoxic drug to a monoclonal antibody, have been developed to enhance the tumor selectivity of anti-cancer payloads [1]. To date, two ADCs, Adcetris (Seattle Genetics/Takeda [2]) and Kadcyla (Roche/ImmunoGen [3]), have been approved for clinical use and over 50 are in development. However, recent data indicate that ADCs cause side-effects that frequently occur before the drugs have reached their appropriate therapeutic dose [4]. Moreover, side-effects caused by the expression of antigen on normal tissue are particularly detrimental and difficult to overcome using conventional ADC technology.

Alternatively, photodynamic therapy (PDT) utilizes a photosensitizing agent in association with the physical energy of non-ionizing light to exert cytotoxic effects via the induction of vascular occlusion at the irradiation site [5]. This method has had some success. However, it is limited by the tumor selectivity of the photosensitizing agent. Because the photosensitizing agents currently in use are often not specific enough, some side-effects, such as normal tissue damage, have been observed [5].

Although ADCs and PDT alone may require further development to avoid their associated side-effects, their combination, designated photoimmunotherapy (PIT), was recently reported as a new type of tumor-targeted therapy [6]. PIT employs a targeted monoclonal antibody-photon absorber conjugate (APC) which improve the selectivity of PDT and directs cytotoxic agents only to the targeted tumor cells in an antigen-dependent manner [7, 8]. Thus, PIT reduces the side-effects of PDT. Furthermore, PIT shows cytotoxicity only after irradiation, meaning that PIT also avoids the side-effects caused by antigen expression on normal tissues which are observed in ADC treatment. Therefore, PIT appears to overcome the major drawbacks of both ADC and PDT simultaneously, making it a valuable tool in cancer treatment.

Epithelial cell adhesion molecule (EpCAM) is a type I transmembrane glycoprotein with a molecular weight of 40 kDa that is highly expressed on human carcinomas [9]. As a proto-oncogene, EpCAM mediates cell adhesion as well as proliferation and signal transduction [10–12]. Notably, EpCAM has been selected as a target antigen for various anti-cancer antibody therapeutics, such as adecatumumab, edrecolomab, 3622W94, and ING-1 [13]. According to clinical reports, both the high-affinity anti-EpCAM antibodies, ING-1 and 3622W94, cause acute pancreatitis, while the other low-affinity anti-EpCAM antibodies, adecatumumab and edrecolomab, produced neither pancreatic side-effects nor any anti-tumor effects [14]. These results suggest that conventional anti-EpCAM antibodies cannot mediate their anti-tumor effects without causing side-effects owing to the expression of EpCAM on pancreas cells. However, these EpCAM antibodies have never been used in conjunction with PIT, which could mediate cytotoxicity in the EpCAM positive cancer cells without inducing pancreatic side-effects.

In the present study, we utilized site-specific conjugation technology [15] to develop a novel APC that targets EpCAM (EpCAM-IR700) for use as an anti-cancer therapy. To our knowledge, this is the first report using this IgG format EpCAM-IR700 PIT method. The efficacy of this method was evaluated both *in vitro* and *in vivo*. Unlike previous reports, which suggest that PIT has antigen-dependent cytotoxicity with limited side-effects [12, 13], our evaluation implies that PIT also induces non-targeted vascular occlusion similar to that observed for PDT.

## RESULTS

### Preparation of the site-specific APCs

**Figure 1:**
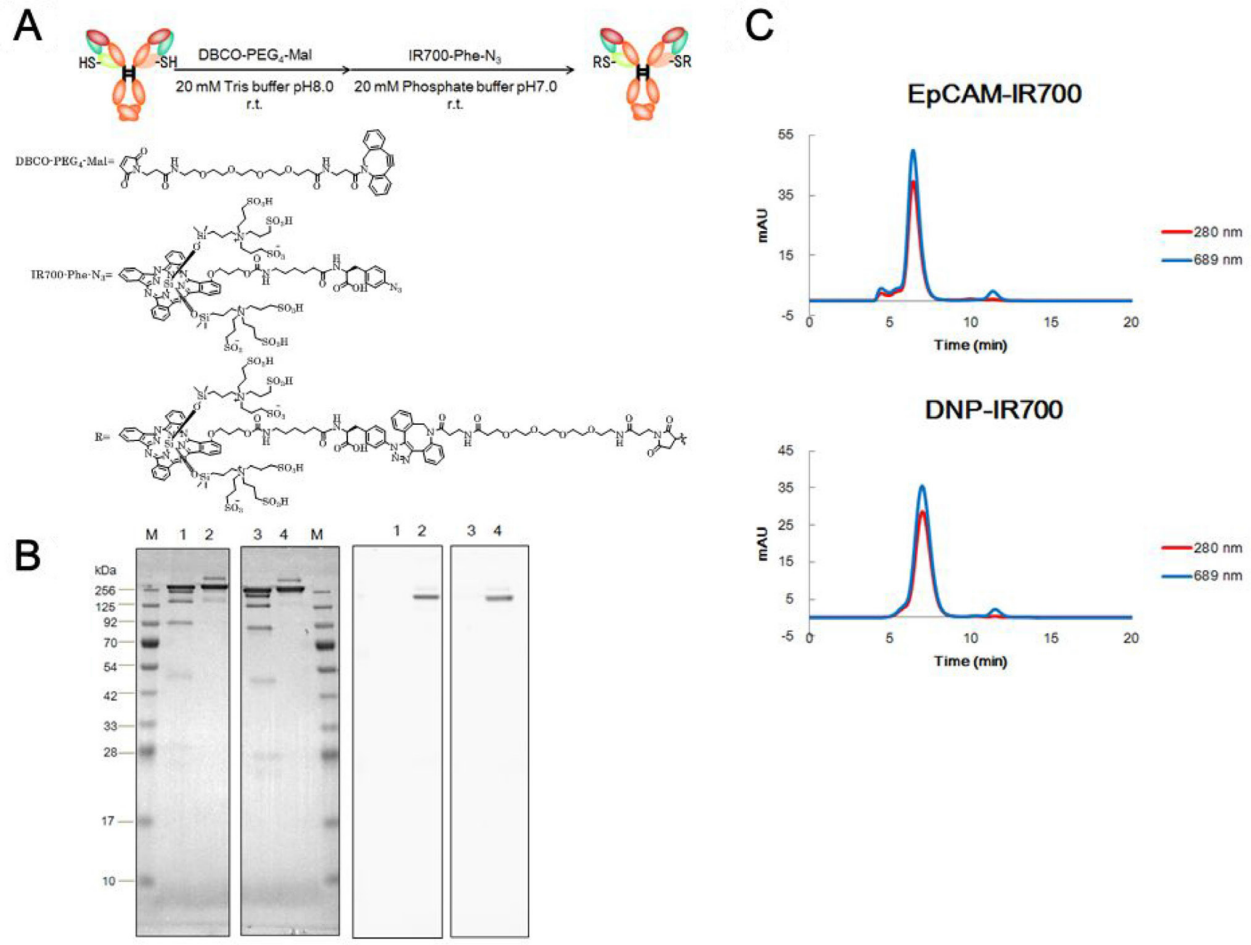
Preparation of site-specific APCs (**A**) A scheme explaining “Actibody” technology and the method used to conjugate the “Actibody” and the IR700 derivative. (**B**) Non-reducing SDS-PAGE analysis of the purified APCs (left: Coomassie Brilliant Blue staining; right: fluorescence detection). Lane M: molecular mass marker; lane 1: anti-EpCAM mAb; lane 2: EpCAM-IR700; lane 3: anti-DNP mAb; lane 4: DNP-IR700. (**C**) Size exclusion chromatography (SEC) analysis of the purified APCs. The 280/494 nm absorbances detected for the elution fractions are shown.

EpCAM-IR700 and anti-2,4-dinitrophenol (DNP)-IR700 (negative control) were prepared with a two-step conjugation method as described in the Materials and methods section (Figure 1A). The drug-to-antibody ratio (DAR) of the prepared APCs was determined using their absorbance in association with the molar extinction coefficient of IR700 and the antibodies. It appears that the DARs of EpCAM-IR700 and DNP-IR700 were 2.16 and 2.05, respectively. Furthermore, both of the APCs showed the expected band sizes in our sodium dodecyl sulfate polyacrylamide gel electrophoresis (SDS-PAGE) analysis. The main band present on the gels also had IR700 fluorescence (Figure 1B). A similar result was observed in our size exclusion chromatography (SEC) analysis, where the main peaks were at 280 nm and 689 nm absorbances (Figure 1C). The retention time of the main peaks for both EpCAM-IR700 and DNP-IR700 were almost identical to that of naked IgG.

### EpCAM-IR700 specifically targets and destroys EpCAM-positive cancer cells *in vitro*

**Figure 2:**
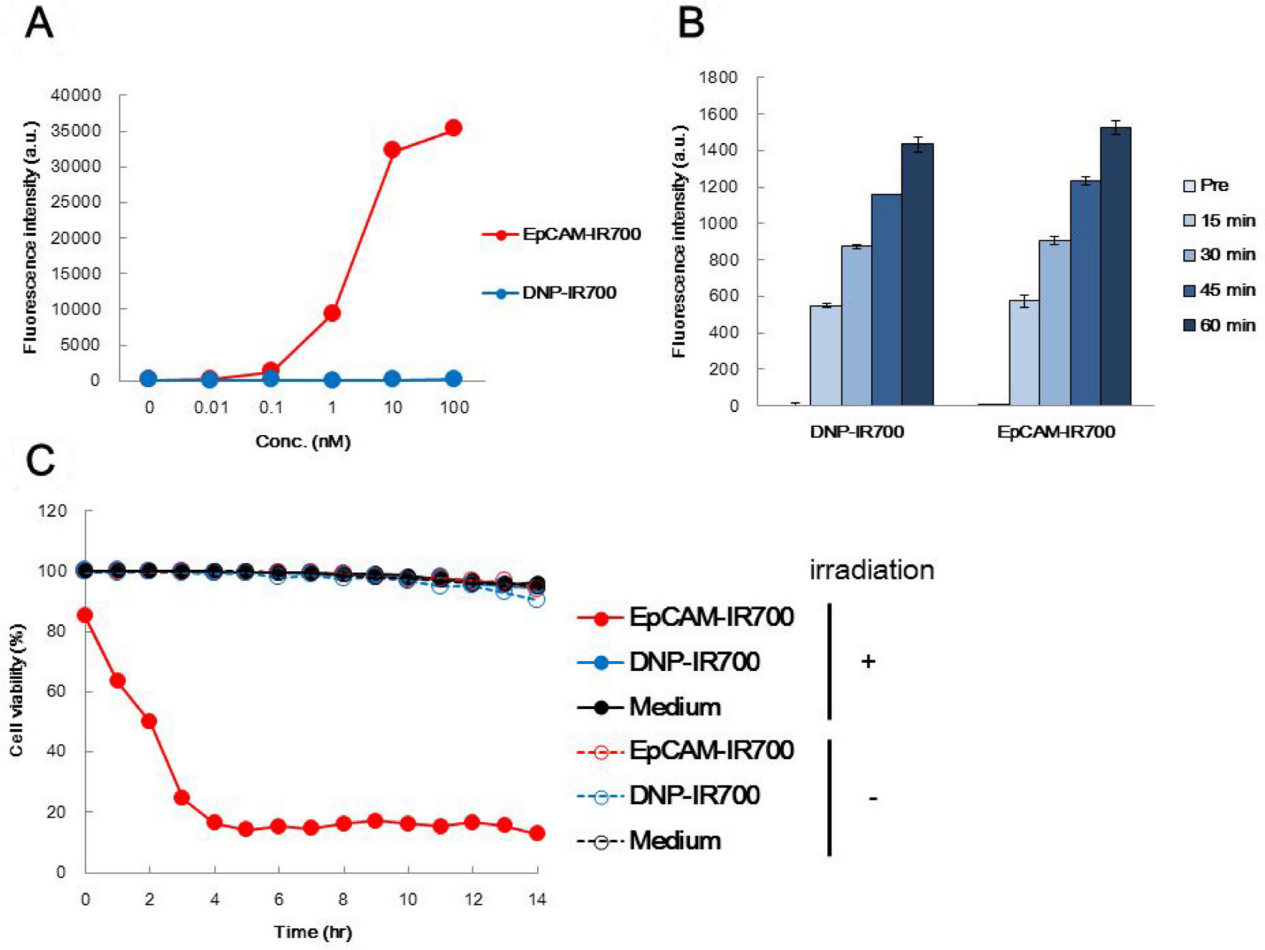
The effects of EpCAM-IR700 and DNP-IR700 *in vitro* (**A**) Flow cytometric analysis of the antigen binding affinity of EpCAM-IR700 (red) or DNP-IR700 (blue) in COLO 205 cells using a FITC-conjugated anti-human IgG detection antibody. (**B**) Analysis of singlet oxygen production induced by each APC using the fluorescence intensity of singlet oxygen sensor green (SOSG) over time. (**C**) The cytotoxicity of EpCAM-IR700 (red) or DNP-IR700 (blue) or medium (black) with or without photoirradiation (PDT; filled in or empty, respectively) in COLO 205 cells was detected over time using 7AAD and Hoechst dye.

The binding capabilities of the EpCAM-IR700 and DNP-IR700 APCs to EpCAM-positive COLO 205 cells was analyzed by flow cytometry. Notably, EpCAM-IR700 treatment increased the fluorescence intensity in an antibody concentration-dependent manner, while DNP-IR700 treatment resulted in almost no fluorescent signal (Figure 2A).

The main mechanism of cytotoxicity in PIT has been reported to involve reactive oxygen species (ROS) [6]. One such ROS is singlet oxygen, which we measured for each APC with singlet oxygen sensor green (SOSG), which emits fluorescence in the presence of this ROS. After irradiation, the SOSG-mediated fluorescence was similar for EpCAM-IR700 and DNP-IR700 (Figure 2B). A cell death assay was also used to measure cytotoxicity. Notably, while cell death was observed in the COLO 205 cells incubated with EpCAM-IR700, this occurred only after irradiation. Almost no cell death was observed in cells incubated with DNP-IR700 (Figure 2C). These results suggest that EpCAM-IR700 specifically destroys EpCAM-positive tumor cells *in vitro*.

### Both EpCAM-IR700 and DNP-IR700 reduce tumor volume *in vivo* after irradiation

**Figure 3:**
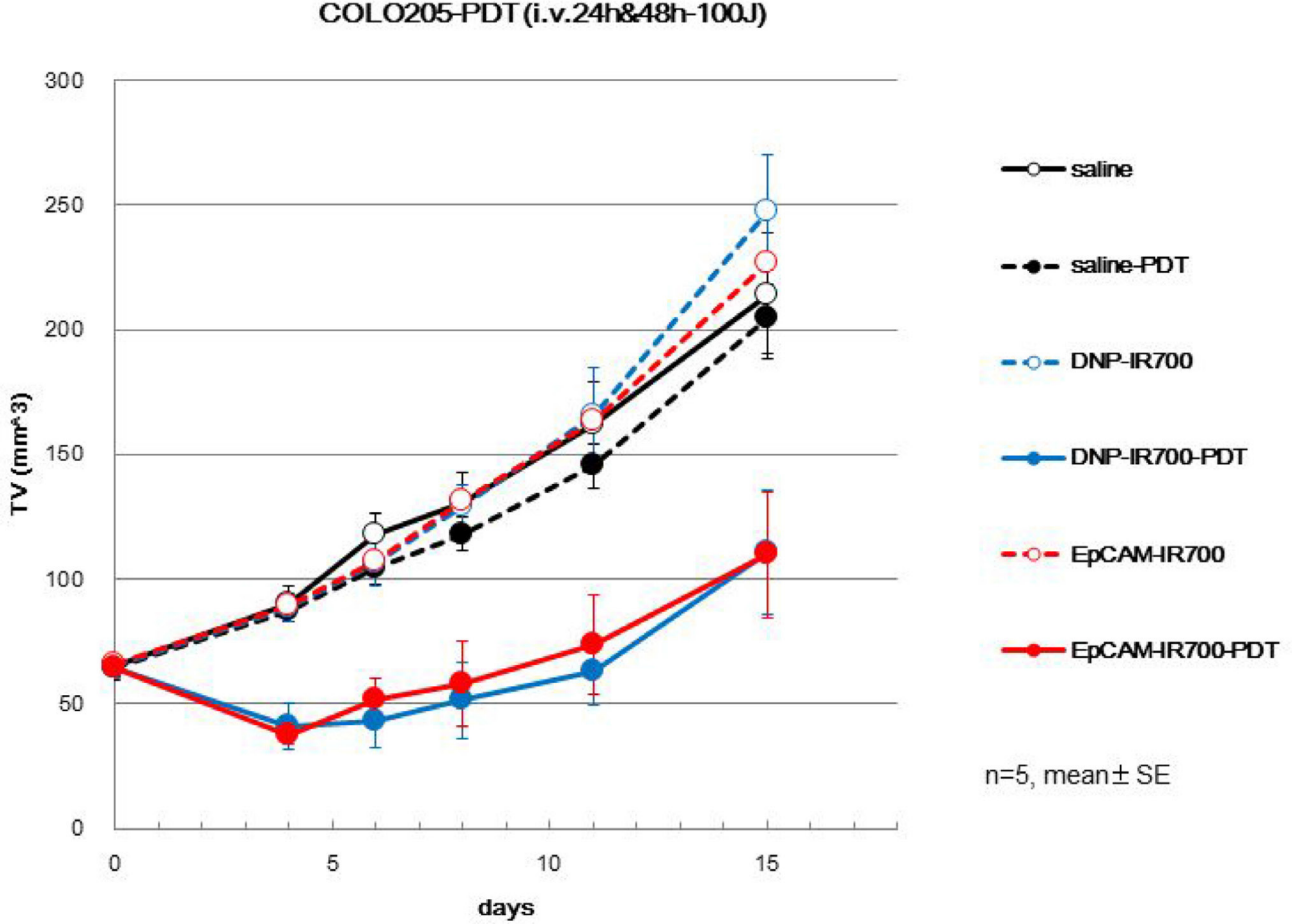
Anti-tumor effects of each APC *in vivo* COLO 205 cells were injected into the left and right flanks of athymic nude mice. After sufficient tumor growth, the mice were intravenously administered EpCAM-IR700 (red), DNP-IR700 (blue), or saline (black), with or without irradiation (PDT; filled in or empty, respectively). Tumor growth inhibition in response to APC treatment with/without irradiation was monitored over time.

To analyze the effects of EpCAM-IR700 and DNP-IR700 *in vivo*, we used tumor-bearing mice implanted with COLO 205 cells. After irradiation, tumor volume was significantly reduced in COLO 205 tumors treated with EpCAM-IR700 (Figure 3). Interestingly, similar results were observed for DNP-IR700. This is in contrast with our *in vitro* analysis.

### *In vivo* mechanism analysis

**Figure 4:**
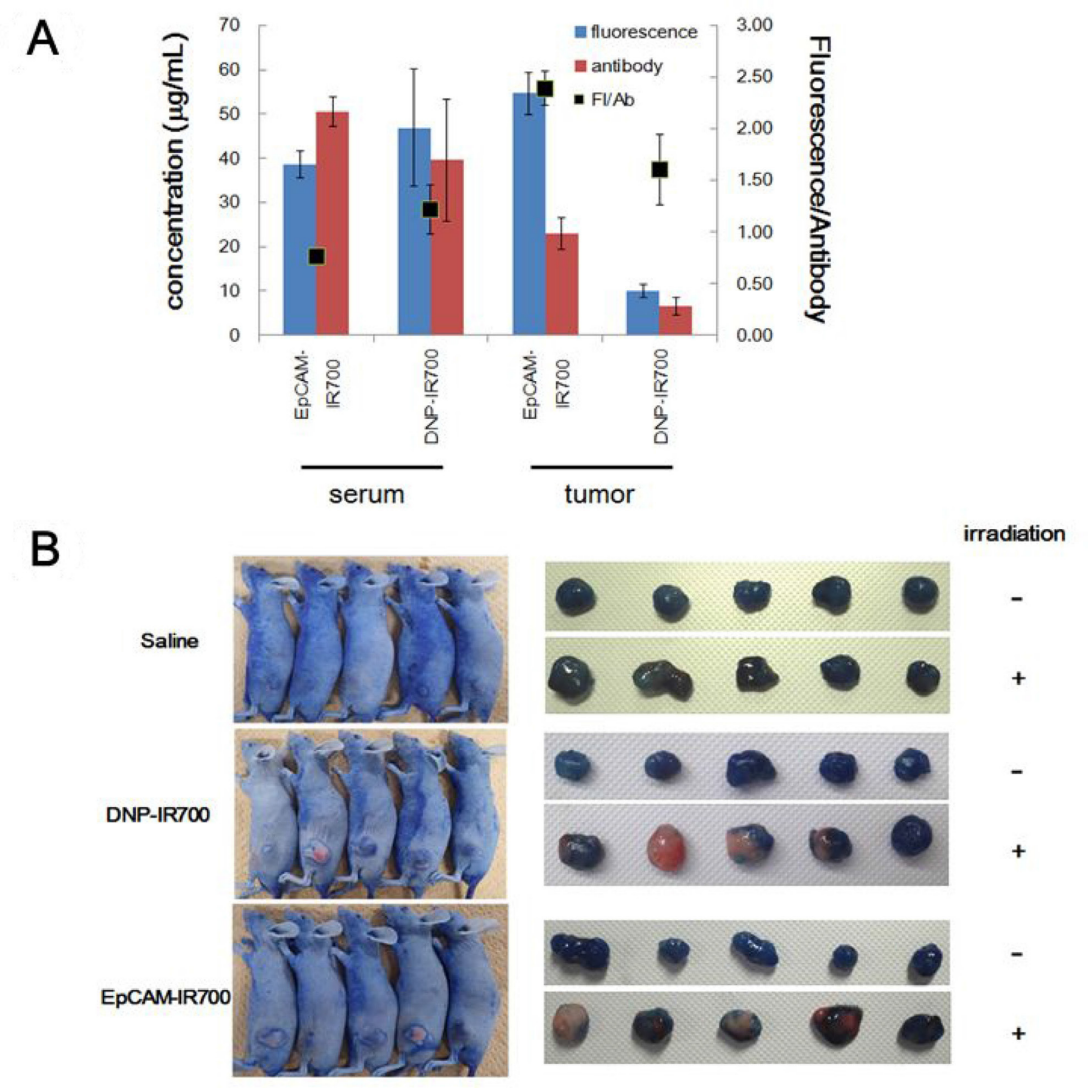
APC-induced vascular occlusion *in vivo* (**A**) Concentration of each APC and payload in tumor tissue and serum samples as well as the fluorescence. (**B**) Images of mice and tumors that received EpCAM-IR700, DNP-IR700, or saline with/without irradiation followed by Evans blue dye extravasation.

To clarify the mechanism underlying the discrepancy between our *in vitro* and *in vivo* analyses, two experiments were conducted. First, we measured APC concentration in the tumors and serum. Our results indicate that the fluorescence intensity as well as the antibody concentration were higher for EpCAM-IR700 than for DNP-IR700 in tumor tissue, whereas they were almost the same in serum (Figure 4A). This suggests that EpCAM-IR700 specifically accumulates in the EpCAM-positive tumor, while DNP-IR700 does not.

Second, we then confirmed vascular occlusion, which is the main mechanism of conventional PDT. This analysis was performed using an Evans blue dye extravasation assay. Our data indicate that both EpCAM-IR700 and DNP-IR700 treatment decreased Evans blue accumulation in the tumors after irradiation compared with non-irradiated tumors (Figure 4B). This implies qualitatively that the IR-700-conjugated antibodies non-specifically damage tumor cells via vascular occlusion.

## DISCUSSION

Cancer treatments are continuously evolving. Two known treatments, ADCs and PDT, have been widely studied and are in some cases, successful. However, ADC treatment has undesired side-effects caused by the expression of the target antigen on normal tissue, whereas the primary drawback of PDT is its insufficient tumor selectivity, which results in non-specific vascular occlusion of both normal and cancerous tissue at the irradiation site [5]. To overcome these issues, PIT was developed as a new type of targeted therapy that combines both APC and PDT technologies. PIT uses a targeted APC that improves the selectivity of PDT, meaning that antigen-specific cytotoxicity can be directed to the targeted cells [6]. Furthermore, PIT-induced cytotoxicity occurs in a light-dependent manner, thus avoiding the aberrant effects on normal tissues observed for conventional ADC treatments. Because of these characteristics, PIT is expected to simultaneously overcome the drawbacks of PDT and ADC by improving tumor selectivity of the photosensitizing agent as well as controlling cytotoxicity with light. As this method is still relatively new, it has not been tested for a wide range of targets. In this study, we developed a novel APC targeting EpCAM for use with PIT (EpCAM-IR700). Both this and the negative control APC (DNP-IR700) were monomer IgG format antibodies, with a preciously controlled photon absorber-to-antibody ratio of two. To our knowledge, this is the first report of the development of an IgG format APC with site-specific conjugation technology.

To determine the efficacy of our APC, we performed a range of analyses, both *in vitro* and *in vivo*. For example, singlet oxygen was generated by both the EpCAM APC as well as the negative control, but only after irradiation. Furthermore, our *in vitro* study showed that EpCAM-IR700, but not DNP-IR700, specifically bound to EpCAM-expressing COLO 205 cells and induced cell damage after irradiation. This EpCAM specificity in targeting cancer cells *in vitro* is supported by other reports demonstrating similar antigen-dependent targeting using this membrane protein [6]. In contrast to this previous study, in our *in vivo* anti-tumor analyses using a mouse xenograft model, we observed significantly reduced tumor volume after irradiation in both the EpCAM-IR700-treated mice as well as the DNP-IR700-treated mice. This discrepancy between this and the previous study was surprising as almost the same experimental procedure was conducted. Moreover, the mechanism underlying these *in vivo* anti-tumor effects was shown to be vascular occlusion. Therefore, these data indicate that unlike our *in vitro* findings, PIT using this EpCAM-targeting APC might induce non-specific anti-cancer effects *in vivo*.

Notably, our results also differed from previous reports investigating APCs targeting two epidermal growth factor receptors, EGFR-IR700 [7] and HER2/Tra-IR700 [8]. In fact, EGFR-IR700 was shown to selectively destroy EGFR-positive A431 cells in the presence of EGFR-negative cells in a mixed tumor model [7], while adenoviral infection with Tra-IR700 destroyed only HER2-expressing cancer cells with minimal cytotoxicity to non-infected cells [8]. It is important to note that in both cases, the experimental conditions, such as target cells and *in vivo* model, were different from those used in the present study and could cause the observed discrepancies. Blood vessel volume as well as blood flow in EGFR-positive cells might also be greater than that in EGFR-negative cells because EGFR signaling induces angiogenesis [16], thus increasing exposure of the cells to the APCs and allowing a greater number of EGFR-positive cells to be destroyed. With regards to the *in vivo* models, these previous studies used a peritoneal dissemination model and i.p. administration, which presumably mimic the *in vitro* conditions as APC accumulation occurs in a closed space. However, in our study, the subcutaneous transplant model used to observe the *in vivo* anti-tumor effects of our APC is highly dependent on the blood vessels. Each of these differences could have resulted in the discrepancies observed between our analyses and those previously reported for other APCs.

The composition of the antibody used for the APC can also potentially affect its function. It was previously reported that a CD25-targeted APC could deplete T regulatory cells distant from the irradiation site without inducing vascular occlusion *in vivo* [17]. In that study, F(ab’)2, which lacks the Fc domain, was used. Notably, the Fc domain binds to the neonatal Fc receptor (FcRn) in the acidic endosomes of vascular endothelial cells and can be recycled back into the blood at physiological pH [18]. Therefore, it is possible that APCs composed of an antibody fragment without this domain could selectively destroy the target cells without inducing vascular occlusion because it cannot bind to FcRn or be kept at low concentrations in the blood. In the present study, the concentration of EpCAM-IR700 was higher than that of DNP-IR700 in tumor tissue, but there was no difference in their blood concentrations, and the blood concentrations appear to be correlated with their *in vivo* anti-tumor effects. Considering these results, the antigen-independent vascular occlusion we observed could be due to the IgG format of the APCs.

In addition to vascular occlusion, tumor-associated macrophage (TAM) depletion by PDT may also contribute to the observed anti-tumor effects *in vivo*. A recent paper reported that the tumor microenvironment, including the presence/absence of immune cells, plays a key role in cancer progression and metastasis [19]. It was also reported that the Fc-FcγR interaction between ADCs and TAMs potentially contributes to the preclinical anti-tumor activities of ADCs in an antigen-independent manner [20]. Unfortunately, analysis of this APC-TAM interaction and the role of the tumor microenvironment in the anti-tumor effects of our novel APCs is beyond the scope of this study and further analysis is required.

In addition, our study was also limited with regards to the PIT irradiation conditions. Bisland *et al.* [21] reported that conventional PDT can be used to exert direct cytotoxicity only to cancers cells without vascular occlusion of the surrounding normal tissue by controlling the irradiation conditions. This implies that appropriate treatment conditions, such as the exposure time, photosensitizer concentration, and light intensity, could be determined and utilized to limit the side-effects of PDT. It is also possible that similar changes and optimization could be applied to PIT. Our results indicate that although PIT is considered to be an antigen-specific anti-tumor therapy, it may induce undesired vascular occlusion in an antigen-independent manner. Hence, control of the treatment conditions could also be important to better direct the cytotoxicity specifically to the tumors cells without vascular occlusion of the normal tissue. Additional studies are required to assess how altering the PIT irradiation conditions could be used to limit the observed side-effects.

In conclusion, we have developed an IgG format APC targeting EpCAM utilizing site-specific conjugation technology (Actibody technology) to be used for cancer treatment in conjunction with PIT. We confirmed the *in vitro* cytotoxic effect of this APC following the experimental conditions used by previous reports. However, we found that PIT might induce vascular occlusion in an antigen-independent manner in our mouse xenograft model. This was surprising as we followed a PIT protocol similar to that used in previous reports, which did not observe vascular occlusion. These discrepancies could be caused by minor differences in the experimental conditions, such as the *in vivo* model, antibody format, irradiation exposure time, APC blood concentration, and light intensity. The use of site-specific conjugation rather than random conjugation may have also affected the results. Furthermore, it is possible that the released payload after degradation could be exerting the non-specific vascular occlusion. To confirm these possibilities, the *in vivo* pharmacodynamics should be further evaluated. Although further study such as the evaluation of vascular density, status of pericytes, and effect on tumor hypoxia is needed to fully understand the anti-cancer mechanism utilized by our EpCAM-targeting APCs, our study highlights significant issues with PIT, which, similar to PDT, involve the possible induction of non-specific cytotoxic activity by vascular occlusion *in vivo*. The experimental conditions require further optimization to minimize the non-specific side effects of this technique, which should be addressed prior to widespread clinical use.

## MATERIALS AND METHODS

### Reagents and cells

IRDye700DX NHS ester (IR700) was purchased from LI-COR Biosciences (Lincoln, NE, USA). The Chinese hamster ovary (CHO) and human colon adeno carcinoma (COLO 205) cell lines were obtained from Thermo Fisher Scientific (Waltham, MA, USA) and American Type Culture Collection (ATCC, Manassas, VA, USA), respectively.

### Cell culture

CHO cells were cultured at 37° C with 5% CO_2_ in FreeStyle CHO expression medium (Thermo Fisher Scientific, Waltham, MA, USA) supplemented with 4 mM L-glutamate (Thermo Fisher Scientific). COLO 205 cells were cultured at 37° C with 5% CO_2_ in RPMI1640 (Thermo Fisher Scientific) supplemented with 10% heat-inactivated fetal bovine serum and 50 μg/mL gentamicin (Nacalai, Kyoto, JAPAN) or penicillin/streptomycin (final concentrations 100 U/mL and 100 μg/mL, respectively; Nacalai Tesque, Kyoto, Japan).

### Construction, expression, and purification of actibody

The cDNAs encoding the heavy- and light-chain variable regions of the anti-EpCAM antibody (clone 323/A3) [22] and the DNP antibody, which was internally produced previously [23], were cloned into an mammalian expression vector. This vector encodes IgG1-Fc, which contains a cysteine mutation enabling actibody production in mammalian cells [15]. These constructed vectors were introduced into CHO cells using the FreeStyle™ MAX CHO Expression System (Thermo Fisher Scientific). Purification of each actibody, respectively, named EpCAM-Lc-Q124C and DNP-Lc-Q124C, from the culture supernatant was performed as previously reported [15].

### Synthesis of the IR700-derived linker

IRDye700DX NHS ester (1.5 mg, 0.77 mmol), (S)-2-amino-3-(4-azidephenyl)propanoic acid (4 mg, 19 mmol), and pyridine (1.6 ml, 19 mmol) were dissolved in N,N-dimethylformamide. After stirring overnight at room temperature, the reaction mixture was filtered and purified by preparative HPLC. Elution was performed with a gradient of 0–90% acetonitrile/THF=1/1–50 mM ammonium acetate for 30 min. The collected fractions were pooled and immediately frozen prior to lyophilization, subsequently producing the linker IR700-Phe-N_3_ (yield 1.5 mg, 99%; ESI-MS (m/z), [M]^-^ for C_79_H_105_N_15_O_26_S_6_Si_3_, 1957.40; calculated, 1956.02).

### Preparation of APCs

EpCAM-IR700 and DNP-IR700 were prepared using a two-step conjugation method. First, conjugation was achieved using a PEG4 linker containing a maleimide linker (DBCO-PEG4-Mal) (Merck, St. Louis, MO, USA). This occurs via the functional sulfhydryl group of the cysteine residues in the actibody. Conjugation of the IR700 derivative (IR700-Phe-N3) linker was achieved using copper-free click chemistry (Figure 1A). After conjugation, EpCAM-Lc-Q124C and DNP-Lc-Q124C were both adjusted to 70 mM with 20 mM phosphate buffer (pH 7.0). They were then conjugated with 25-fold molar excess of DBCO-PEG4-Mal in the presence of 16.7% v/v DMSO solution at 4° C overnight. After eliminating the excess DBCO-PEG-Mal using NAP-5 (GE Healthcare) and Amicon Ultra 50K (Merck), the purified EpCAM-DBCO-PEG4-Mal and DNP-DBCO-PEG4-Mal were adjusted to 100 mM with 20 mM phosphate buffer (pH 7.0) and conjugated with 10-fold molar excess of IR700-Phe-N3 at 4° C overnight. After conjugation, EpCAM-IR700 and DNP-IR700 were purified and the excess IR700-Phe-N3 was removed via buffer-exchange with 20 mM citrate buffer (pH 6.0) as described above. The purity of each APC was examined by sodium dodecyl sulfate polyacrylamide gel electrophoresis (SDS-PAGE) and size exclusion chromatography (SEC) analyses. The DAR of the APCs was checked using their absorbance in association with the molar extinction coefficients of IR700 and the antibodies.

### Measurement of APC activity *in vitro*

The antigen-binding capabilities of each APC were measured in COLO 205 cells using flow cytometry. COLO 205 cells were cultured and stained with each APC at 4° C for 30 min. Fluorescein isothiocyanate (FITC)-conjugated anti-human IgG(H+L) (R&D Systems, Minneapolis, MN, USA) was used as the secondary antibody to detect the cell-bound APCs. The stained cells were then analyzed with a BD FACSVerse (BD Biosciences, San Jose, CA, USA).

APC-mediated generation of singlet oxygen was also confirmed using singlet oxygen sensor green (SOSG) (Thermo Fisher Scientific), which is highly selective and emits fluorescence in the presence of singlet oxygen. For this analysis, 100 nM of each prepared APC was mixed with SOSG and irradiated with near infrared (NIR) light (690 nm, 28.7 mW) for 15 min. After irradiation, the fluorescence was measured with a spectrofluorometer using an excitation/emission of 488/525 nm. The same procedure was repeated three times.

The cytotoxic activity of the APCs was evaluated with COLO 205 cells using an IN Cell Analyzer 6000. COLO 205 cells were incubated with 100 nM of each APC at 4° C for 1 h. The cells were then washed with phenol-red free D-MEM (Thermo Fisher Scientific), and fresh D-MEM containing 7-amino-actinomycin D (7AAD) (BD Biosciences) and Hoechst 33342 solution (DOJINDO, Kumamoto, Japan) were added. After NIR irradiation (690 nm, 28.7 mW) for 3 min, cell viability was determined based on 7AAD uptake and Hoechst staining.

### Evaluation of APC anti-tumor effects *in vivo*

All animal studies were performed in accordance with the Standards for Proper Conduct of Animal Experiments at Kyowa Hakko Kirin Co., Ltd. under the approval of the company’s Institutional Animal Care and Use Committee (protocol number APS15J0233). The Fuji Research Park/Tokyo Research Park of Kyowa Hakko Kirin Co., Ltd. is fully accredited by the Association for the Assessment and Accreditation of Laboratory Animal Care, International.

Athymic nude mice (BALB/cAJcl-nu/nu, female, 5 weeks old) were purchased from CLEA Japan, Inc. (Tokyo, Japan). The animals were maintained under specific pathogen-free conditions with free access to autoclaved tap water and irradiated feed (CL-2, CLEA Japan, Inc.). COLO 205 cells (5 × 10^6^ cells/0.05 mL) suspended in Dulbecco’s phosphate buffered saline (PBS, Invitrogen) were subcutaneously implanted in both the right and left sides of each mouse. The resulting tumors were measured 9 days after cell implantation using calipers, and the tumor volumes were calculated with the following formula: tumor volume = length × width × width × 0.5. Mice with tumors ranging from approximately 52 to 80 mm^3^ were selected and divided into three groups (*n* = 5 mice/group) with comparable mean tumor volumes. The day of grouping was set as day 0. IR700 conjugated APCs (300 μg) were administered to each mouse intravenously starting at day 0. Furthermore, the tumors in their left flanks were exposed to 100 J/cm^2^ of NIR light on days 1 and 2. For NIR irradiation, light-emitting diode (LED) lights (L690-66-60, Marubeni America Co., Santa Clara, CA), which emit light at 670–710 nm (peak at 690 nm), were used at approximately 44 mW/cm^2^. The power densities were measured with an optical power meter (PM 100, Thorlabs, Newton, NJ, USA). During NIR exposure, mice were anesthetized with isoflurane. Tumor volume and body weight was measured two times a week.

### Vascular occlusion test

The blood flow in the subcutaneous COLO 205 tumor vasculatures was determined using Evans blue dye. Tumor-bearing mice were divided into four groups (*n* = 5 mice/group) with comparable mean tumor volumes and were administered saline (control), DNP-IR700, EpCAM-IR700, or Pani-IR700 at a dose of 300 μg/mouse. After treatment (24 h), the tumors were exposed to 100 J/cm^2^ of NIR light. During the second round of irradiation on the following day, the animals were also intravenously injected with Evans blue (2.5% in PBS, Sigma, St. Louis, MO). Approximately 10 min after injection, the tumor-bearing animals were euthanized, and the tumor tissues were excised and observed.

### Analysis of APC concentration in blood and tumor tissue

Tumor-bearing mice were divided into four groups (n = 3 mice/group) with comparable mean tumor volumes and were treated with DNP-IR700, EpCAM-IR700, or Pani-IR700 at 300 μg/mouse. The mice were euthanized 24 h later, and blood and tumor tissue samples were taken, following to the previous paper [24]. Blood samples were left at room temperature, and the serum was obtained by centrifugation (room temperature, 8000 rpm, 10 min). The serum samples were stored at <–20° C until analysis. The tumor samples were immediately frozen with liquid nitrogen. A 4´ volume of NP40 Cell Lysis Buffer (Life technologies, FNN0021) and a 5-mm zirconia bead (AsOne, YTZ-0.5) were added to the tumor sample, followed by homogenization with a Tissue Lyser II (QIGEN). Crude homogenates were centrifuged (4° C, 10000 rpm, 5 min), and the supernatants were collected. The tumor samples were stored at <–80° C.

### Electrochemiluminescent immunoassay

To determine the total antibody concentration in the serum and tumor tissue samples, we used an electrochemiluminescent immunoassay. Blocking buffer (PBS containing 1% w/v casein) was added to each well of a 96-well plate (MULTI-ARRAY 96-well Streptavidin Plate, Meso Scale Discovery) and incubated at room temperature for 1 h. After the solution was discarded, the plate was washed three times with wash buffer (PBS containing 0.05% v/v Tween 20). Then, capture antibody (biotinylated anti-human IgG) was added to each well and incubated for 1 h at room temperature. After washing, calibration standards and analytical samples were added to each well and incubated for an additional 2 h at room temperature. The plate was washed three times, followed by the addition of detection antibody (ruthenylated anti-human IgG). After incubation for 1 h at room temperature, the plate was washed three times. Finally, read buffer T (R92TC-1, Meso Scale Discovery) was added to each well, and the electrochemiluminescent signals were detected using a SECTOR Imager 2400 (Meso Scale Discovery). A calibration curve was generated by log-log regression, except for the 0 ng/mL sample, using SOFTmax^®^ PRO (Nihon Molecurar Devices). The concentration of each APC was calculated by substituting the signal intensity into the regression equation for each calibration curve.

### Determination of total IR700 fluorescence

We utilized the inherent fluorescence of IR700 to determine its total concentration in the serum and tumor samples. Analytical samples and calibration standards were added to each well of a 384-well plate (CELLSTAR μClear 384 well microplate, Greiner Bio-One). The fluorescence intensity was measured with a SpectraMax M5 (excitation/emission 684/702 nm, cutoff 695 nm; Molecular devices). A calibration curve was generated via linear regression, except for the 0 ng/mL sample. The total IR700 concentration for each APCs was calculated by substituting the signal intensity into the calculated regression equation for each calibration curve.
